# Antihemolytic Activities of Green Tea, Safflower, and Mulberry Extracts during *Plasmodium berghei* Infection in Mice

**DOI:** 10.1155/2014/203154

**Published:** 2014-11-18

**Authors:** Suthin Audomkasok, Waraporn Singpha, Sukanya Chachiyo, Voravuth Somsak

**Affiliations:** Department of Clinical Chemistry, Faculty of Medical Technology, Western University, Kanchanaburi 71170, Thailand

## Abstract

Malaria-associated hemolysis is associated with mortality in adult patients. It has been speculated that oxidative stress and inflammation induced by malaria parasite are involved in its pathophysiology. Hence, we aimed to investigate the antihemolytic effect of green tea, safflower, and mulberry extracts against *Plasmodium berghei* infection. Aqueous crude extracts of these plants were prepared using hot water method and used for oral treatment in mice. Groups of ICR mice were infected with 6 × 10^6^ infected red blood cells of *P. berghei* ANKA by intraperitoneal injection and given the extracts (500, 1500, and 3000 mg/kg) twice a day for 4 consecutive days. To assess hemolysis, hematocrit levels were then evaluated. Malaria infection resulted in hemolysis. However, antihemolytic effects were observed in infected mice treated with these extracts at dose-dependent manners. In conclusion, aqueous crude extracts of green tea, safflower, and mulberry exerted antihemolysis induced by malaria infection. These plants may work as potential source in the development of variety of herbal formulations for malarial treatment.

## 1. Introduction

Malaria is an enormous public health problem worldwide, especially tropical and subtropical area, and kills 730,000 people annually mostly children residing in Africa. It is caused by the parasite* Plasmodium* and transmitted by the bite of* Anopheles* mosquito [1]. Malaria-associated acute hemolysis, one of the major life-threatening well-known causes of death in* P. falciparum* and* P. vivax*, occurs between 1 and 4% of hospitalized patients with a mortality that can be up to 45% [2, 3]. The pathogenesis of malarial-associated acute hemolysis has suggested involvement of cytoadherence of infected red blood cell (RBC) and inflammatory response as well as oxidative stress through generation of reactive oxygen intermediates by host cells [4, 5]. Moreover, parasite invasion and subsequent RBC rupture also contributed to pathogenesis of hemolysis. This has prompted research towards the discovery and development of new, safe, and affordable antihemolysis drugs during malaria infection. In this respect, medicinal plants are potential targets for research.

Recently, interest in green tea (*Camellia sinensis*), safflower (*Carthamus tinctorius*), and mulberry (*Morus alba*) as promising agents for the prevention or reduction of risk for many human diseases involving oxidative stress has increased. They are popular beverage worldwide which contain large amounts of polyphenols and flavonoids. The possible beneficial effects of these tea extracts in the prevention of cancer as well as cardiovascular, neurodegenerative, and other diseases have been studied extensively [6–9]. With regard to preventing hemolysis, the antioxidant activity of these tea extracts might play an important role. In this study, we aimed to evaluate the effects of green tea, safflower, and mulberry extracts on hemolysis during* P. berghei* infection in mice.

## 2. Materials and Methods

### 2.1. Plant Materials and Preparation of Crude Extracts

Commercial dried leaves of green tea (*Camellia sinensis*), safflower (*Carthamus tinctorius*), and mulberry (*Morus alba*) were obtained from Royal Project Foundation shop, Chiang Mai, Thailand. The voucher specimen has been deposited in the Department of Pharmacology, Faculty of Medicine, Chiang Mai University, Chiang Mai, Thailand. The plants were air-dried at room temperature and subsequently powdered. Dried leaves of plants (10 g) were used to prepare aqueous crude extracts with 100 mL of distilled water using hot water method [10]. The crude extracts contained >60% of total polyphenols, flavonoids, gallic acid, epigallocatechin, 4-hydroxybenzhydrazide derivative, and <0.1% of caffeine by HPLC.

### 2.2. Experimental Animal

ICR mice, 6–8 weeks old, weighing 30–35 g purchased from National Laboratory Animal Center, Mahidol University, Bangkok, Thailand, were used in this study. They were housed in 12 h light/12 h dark cycle with 22–25°C and given standard mouse pellet diet and clean water* ad libitum*. All animal experiments were approved by the Ethical Committee on Animal Experimentation, Faculty of Medical Technology, Western University, Kanchanaburi, Thailand.

### 2.3. Rodent Malaria Parasite

Chloroquine-sensitive* Plasmodium berghei* strain ANKA (PbANKA) was used. The parasite was kept alive by continuous intraperitoneal (IP) passage in mice. Blood was collected from tail vein and parasitemia was daily monitored by microscopic examination of Giemsa stained thin blood smear.

### 2.4. Measurement of Hematocrit Levels

Percent hematocrit (% Hct) was measured by collecting of tail blood into heparinized capillary tube and centrifugation at 10,000 g for 10 min. Proportion of packed RBC and total blood volume was finally calculated.

### 2.5. Efficacy Test of Tea Extracts* In Vivo*

The experiment of* in vivo* test was based on standard 4-day suppressive test [11]. Groups of ICR mice (5 mice of each) were inoculated with 6 × 10^6^ infected RBC of PbANKA by IP injection. They were then treated orally twice a day for 4 consecutive days with 500, 1500, and 3000 mg/kg of the extracts. Three control groups were used; normal controls were treated either distilled water or extracts while the untreated control was given only distilled water. On day 5 of the experiment, tail blood was collected and measured % Hct.

### 2.6. Statistical Analysis

The results were expressed as mean ± standard deviation (SD). Significant difference was assessed by one-way ANOVA or, when appropriate, Student's *t*-test for paired observation. Values of *P* < 0.05 were considered significant.

## 3. Results

### 3.1. Malaria-Associated Hemolysis Induced by* Plasmodium berghei* Infection

**Figure 1:**
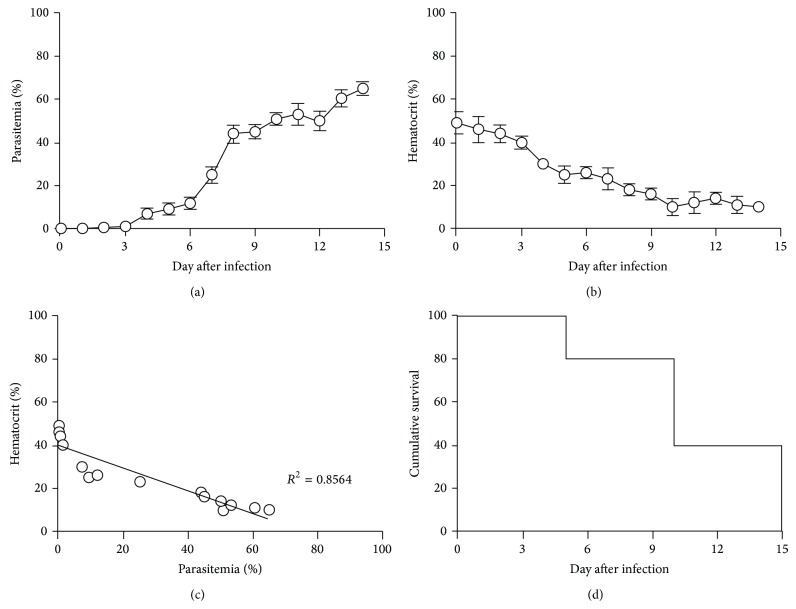
Malaria-associated hemolysis induced by* Plasmodium berghei* infection. ICR mice (5 mice of each) were inoculated by 6 × 10^6^ infected RBC of PbANKA by IP injection. (a) Parasitemia and (b) hematocrit levels were daily monitored. (c) Correlation of parasitemia and hematocrit and (d) cumulative survival of infected mice were also determined. Results were expressed as mean ± SD.

Parasitemia was first detectable on day 3 after infection with a parasitemia of 0.5% and reached to 65% on day 14 (Figure 1(a)). Next, we observed that % Hct was markedly decreased in infected mice, and the onset of hemolysis came out from day 4 after infection (Figure 1(b)). Additionally, strong negative correlation (*R* ^2^ = 0.8564) between parasitemia and % Hct was also obtained (Figure 1(c)), and the survival time of infected mice was 14 days (Figure 1(d)).

### 3.2. Antihemolytic Effects of Green Tea, Safflower, and Mulberry Extracts during* Plasmodium berghei* Infection in Mice

**Figure 2:**
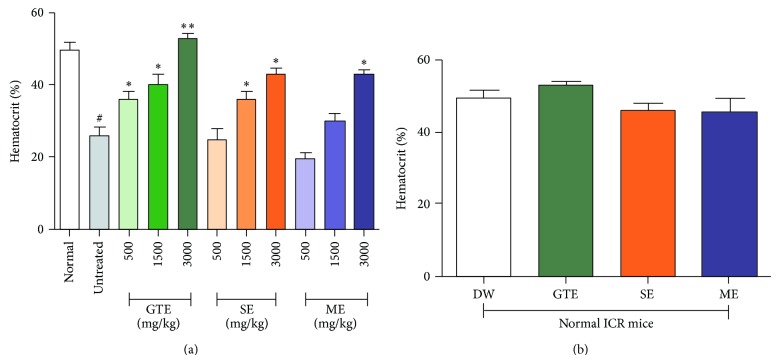
Antihemolytic effects of green tea, safflower, and mulberry extracts against* Plasmodium berghei* infection. Groups of ICR mice (5 mice of each) were inoculated with 6 × 10^6^ infected RBC of PbANKA by IP injection and subsequently given 500, 1500, and 3000 mg/kg of the extracts orally twice a day for 4 consecutive days. On day 5 of experiment, % Hct was measured in (a) infected mice treated with these extracts. In addition, (b) % Hct of normal mice treated with these extracts was also measured. Results were presented as mean ± SD. ^#^ *P* < 0.01 compared to normal; ^*^ *P* < 0.05 and ^**^ *P* < 0.01 compared to untreated control. GTE: green tea extract, SE: safflower extract, and ME: mulberry extract.

The results showed that aqueous crude extracts of green tea, safflower, and mulberry exerted dose-dependent antihemolytic effects against PbANKA infection in mice (Figure 2(a)). These extracts caused a significant effect (*P* < 0.05 and *P* < 0.01) when compared to the untreated control which showed significant (*P* < 0.01) decrease in % Hct, compared to normal mice. The highest antihemolytic activity was found in infected mice treated with green tea, followed by safflower and mulberry extracts. Additionally, there were no effects on % Hct in normal ICR mice treated with these extracts at a maximum dose of 3000 mg/kg (Figure 2(b)).

## 4. Discussion

In this study, we tested the efficacy of green tea, safflower, and mulberry extracts on hemolysis during PbANKA infection in mice. The results showed that during blood stage propagation of PbANKA hematocrit was decreased and infected mice would die from severe anemia. Malaria-associated hemolysis is proposed to be a consequence of parasite development in RBC as well as exacerbated RBC membrane against products of oxidative stress releasing during infection [12]. Moreover, the destruction of RBC during blood stage of infection accumulates high levels of toxic free heme in circulation that, in turn, has the ability to induce oxidative stress from production of hydroxyl radicals via the Fenton/Haber-Weiss reaction [13]. Lipid peroxidation of RBC membrane followed by hemolysis has also been suggested [14]. Additionally, recruitment of inflammation during pathogenesis of malaria-associated hemolysis also contributes to increase the occurrence of hemolytic events [15, 16]. For the efficacy test* in vivo* of these extracts against PbANKA induced hemolysis presented the same level of hematocrit compared with normal control. It can be suggested that polyphenols and flavonoid contents in these extracts might play a central role to protect RBC from oxidative stress and inflammation induced by malaria infection [17]. It has been also reported that polyphenolic contents strongly positive correlated to antioxidant activity in tea extracts [18]. Moreover, malaria can cause metabolic acidosis via RBC destruction followed by severe anemia [19]. Green tea, safflower, and mulberry extracts have been reported to maintain blood pH as well as protect RBC from acidosis [20, 21]. Other mechanisms of action should be searched for.

## 5. Conclusions

Taken together, our results suggest that both aqueous crude extracts of green tea, safflower, and mulberry show antihemolytic activities against* P. berghei*-induced hemolysis. Appropriate pharmaceutical strategies might now be devised to increase the low bioavailability of these plant extracts and to protect them against rapid* in vivo* metabolic transformation, in such a way to make them more amenable as alternative antimalarial drugs in combination therapies.
